# Heterologous Protection against Asian Zika Virus Challenge in Rhesus Macaques

**DOI:** 10.1371/journal.pntd.0005168

**Published:** 2016-12-02

**Authors:** Matthew T. Aliota, Dawn M. Dudley, Christina M. Newman, Emma L. Mohr, Dane D. Gellerup, Meghan E. Breitbach, Connor R. Buechler, Mustafa N. Rasheed, Mariel S. Mohns, Andrea M. Weiler, Gabrielle L. Barry, Kim L. Weisgrau, Josh A. Eudailey, Eva G. Rakasz, Logan J. Vosler, Jennifer Post, Saverio Capuano, Thaddeus G. Golos, Sallie R. Permar, Jorge E. Osorio, Thomas C. Friedrich, Shelby L. O’Connor, David H. O’Connor

**Affiliations:** 1 Department of Pathobiological Sciences, University of Wisconsin-Madison, Madison, Wisconsin, United States of America; 2 Department of Pathology and Laboratory Medicine, University of Wisconsin-Madison, Madison, Wisconsin, United States of America; 3 Department of Pediatrics, School of Medicine and Public Health, University of Wisconsin-Madison, Wisconsin, United States of America; 4 Wisconsin National Primate Research Center, University of Wisconsin-Madison, Madison, Wisconsin, United States of America; 5 Human Vaccine Institute, Duke University Medical Center, Durham, North Carolina, United States of America; 6 Departments of Comparative Biosciences, School of Veterinary Medicine, University of Wisconsin-Madison, Madison, Wisconsin, United States of America; 7 Departments of Obstetrics and Gynecology, School of Medicine and Public Health, University of Wisconsin-Madison, Madison, Wisconsin, United States of America; 8 Department of Pediatrics, Duke University Medical Center, Durham, North Carolina, United States of America; George Mason University, UNITED STATES

## Abstract

**Background:**

Zika virus (ZIKV; *Flaviviridae*, *Flavivirus*) was declared a public health emergency of international concern by the World Health Organization (WHO) in February 2016, because of the evidence linking infection with ZIKV to neurological complications, such as Guillain-Barre Syndrome in adults and congenital birth defects including microcephaly in the developing fetus. Because development of a ZIKV vaccine is a top research priority and because the genetic and antigenic variability of many RNA viruses limits the effectiveness of vaccines, assessing whether immunity elicited against one ZIKV strain is sufficient to confer broad protection against all ZIKV strains is critical. Recently, *in vitro* studies demonstrated that ZIKV likely circulates as a single serotype. Here, we demonstrate that immunity elicited by African lineage ZIKV protects rhesus macaques against subsequent infection with Asian lineage ZIKV.

**Methodology/Principal Findings:**

Using our recently developed rhesus macaque model of ZIKV infection, we report that the prototypical ZIKV strain MR766 productively infects macaques, and that immunity elicited by MR766 protects macaques against heterologous Asian ZIKV. Furthermore, using next generation deep sequencing, we found *in vivo* restoration of a putative N-linked glycosylation site upon replication in macaques that is absent in numerous MR766 strains that are widely being used by the research community. This reversion highlights the importance of carefully examining the sequence composition of all viral stocks as well as understanding how passage history may alter a virus from its original form.

**Conclusions/Significance:**

An effective ZIKV vaccine is needed to prevent infection-associated fetal abnormalities. Macaques whose immune responses were primed by infection with East African ZIKV were completely protected from detectable viremia when subsequently rechallenged with heterologous Asian ZIKV. Therefore, these data suggest that immunogen selection is unlikely to adversely affect the breadth of vaccine protection, i.e., any Asian ZIKV immunogen that protects against homologous challenge will likely confer protection against all other Asian ZIKV strains.

## Author Summary

Zika virus (ZIKV) isolates are genetically diverse, but belong to two recognized lineages, termed “African” and “Asian.” Asian ZIKV infection during pregnancy causes fetal abnormalities including microcephaly. Developing an effective preventative Zika virus vaccine that protects pregnant women is essential for minimizing fetal abnormalities; at least 18 groups are developing ZIKV vaccines [1]. The genetic and antigenic variability of many RNA viruses limits the effectiveness of vaccines, and the degree to which immunity against one ZIKV strain could provide protection against another is unknown. Here we show that rhesus macaques infected with the East African ZIKV strain MR766 are completely protected from subsequent infection with heterologous Asian ZIKV. MR766 is more genetically divergent from all known Asian ZIKV strains than Asian ZIKV strains are from one another. Therefore, ZIKV strain selection is unlikely to compromise vaccine effectiveness.

## Introduction

Zika virus (ZIKV) is an arthropod-borne member of the genus *Flavivirus* of the Spondweni serocomplex that is currently causing an explosive outbreak of febrile disease in the Americas. Historically, ZIKV existed in relative obscurity with only sporadic confirmed human infections until the end of the last century [2]. ZIKV is believed to have originated in Africa, where it is maintained in an enzootic cycle that includes unknown vertebrate hosts (nonhuman primates are suspected) and arboreal *Aedes* mosquitoes [3–5]. In fact, ZIKV was first isolated from the blood of a sentinel rhesus monkey during yellow fever virus surveillance studies in the Zika forest of Uganda [6]. The virus is thought to have spread from East Africa into both West Africa and Asia ~50–100 years ago [7]. Beginning in 2007, ZIKV outbreaks were reported in Yap Island of the Federated states of Micronesia [8], French Polynesia [9], other Pacific islands [10], and in early 2015, in the state of Rio Grande do Norte in northern Brazil [11]. Since its introduction into the Americas, ZIKV has spread essentially uncontrolled with at least 54 countries and territories experiencing autochthonous transmission, including the continental US and multiple US territories [12]. In humans, ZIKV infection typically causes a mild and self-limiting illness known as Zika fever, which often is accompanied by maculopapular rash, headache, and myalgia [13,14]. During the current outbreak, a causal relationship between prenatal ZIKV infection and fetal microcephaly, as well as other serious brain anomalies, has been established [15–17]. Development and testing of vaccines that elicit protective immune responses among girls and women before pregnancy is a top public health priority [18].

The ZIKV genome is an ~11 kb single-stranded, positive sense RNA that contains a single open reading frame. Once the RNA genome is released into the cytoplasm it is directly translated into a polyprotein precursor. The polyprotein is subsequently glycosylated by cellular glycosyltransferases and cleaved by a combination of viral and host proteases to release three structural (C, prM, and E) and seven nonstructural proteins (NS1, NS2A, NS2B, NS3, NS4A, NS4B, and NS5) [19]. The envelope (E) glycoprotein is a target for broadly protective neutralizing antibodies in ZIKV and other flaviviruses and is an attractive candidate immunogen for inclusion in ZIKV vaccines [20]. Understanding the breadth of immunity elicited by the envelope glycoprotein and the host selection of viral variants is therefore important for vaccine design.

There are three distinct genotypes of ZIKV: West African (Nigerian cluster), East African (MR766 prototype cluster), and Asian [21]. All of the ZIKV strains circulating in the Western hemisphere are Asian lineage. The E protein amino acid identity among all Asian lineage ZIKVs is >99%, and as a group, these are only ~96% and 97% amino acid identical to representative East African and West African viruses, respectively [21]. It is not known whether the differences between African and Asian lineage ZIKV have any phenotypic impact, e.g., increased transmissibility or pathogenicity. Because human infections with ZIKV have historically been sporadic, and, until recently, limited to small-scale epidemics, neither the disease caused by ZIKV nor the molecular determinants of immunity have been well characterized. Accordingly, we recently developed an animal model for Asian-lineage ZIKV infection in Indian-origin rhesus macaques (*Macaca mulatta*), and demonstrated that immune responses elicited by infection with Asian ZIKV protected against detectable viremia following rechallenge with the same homologous ZIKV strain [22]. While this demonstrated the potency of naturally elicited antiviral immunity, it did not address whether such immunity is broadly protective against heterologous ZIKV strains.

To investigate the breadth of protective ZIKV immunity between heterologous lineages of the virus, we infected three macaques with the African prototype strain of ZIKV, MR766 [6]. All three animals exhibited an acute, self-limiting infection similar to those previously observed in macaques infected with Asian ZIKV. Immune responses against MR766 provided protection against viremia in all three macaques when re-challenged with an Asian ZIKV strain and dose that productively infected 8/8 naïve macaques.

## Methods

### Study design

This study was designed to examine whether prior infection with African Zika virus (ZIKV) provides protection from heterologous challenge with an Asian ZIKV isolate in the rhesus macaque model. Datasets used in this manuscript are publicly available from http://go.wisc.edu/50bfn2

### Ethical approval

This study was approved by the University of Wisconsin-Madison Institutional Animal Care and Use Committee (Animal Care and Use Protocol Number G005401).

### Animals

Two male and one female, Indian-origin rhesus macaques (*Macaca mulatta*) utilized in this study were cared for by the staff at the Wisconsin National Primate Research Center (WNPRC) in accordance with the regulations, guidelines, and recommendations outlined in the Animal Welfare Act, the Guide for the Care and Use of Laboratory Animals, and the Weatherall report. In addition, all macaques utilized in the study were free of Macacine herpesvirus 1, Simian Retrovirus Type D, Simian T-lymphotropic virus Type 1, and Simian Immunodeficiency Virus. For all procedures, animals were anesthetized with an intramuscular dose of ketamine (10mL/kg). Blood samples were obtained using a vacutainer or needle and syringe from the femoral or saphenous vein. Macaques challenged with Asian ZIKV (Zika virus/H.sapiens-tc/FRA/2013/FrenchPolynesia-01_v1c1; ZIKV-FP) were used for comparison; details on these animals are available in Dudley et al. [22].

### Viruses

ZIKV prototype strain MR766 (referred to as CDC) was obtained from Brandy Russell (CDC, Ft. Collins, CO) and was originally isolated from a sentinel rhesus monkey in 1947 from the Zika Forest, Entebbe, Uganda and passaged 149 times through suckling mouse brains and twice on Vero cells. ZIKV French Polynesian strain (ZIKV-FP) was obtained from Xavier de Lamballerie (European Virus Archive, Marseille, France). It was originally isolated from a 51-year old female in France after travel to French Polynesia in 2013 and passaged a single time on Vero cells. Challenge virus stocks were prepared by inoculation onto a confluent monolayer of C6/36 mosquito cells (ATCC #CRL-1660). A single, clarified harvest of each virus, with titers of 5.9 x 10^6^ PFU/mL (3.9 x 10^9^ vRNA copies/mL) and 1.26 x 10^6^ PFU/mL (1.43 x 10^9^ vRNA copies/mL) for Zika virus/R.macaque-tc/UGA/1947/MR766-3329 (referred to as challenge stock) and ZIKV-FP, respectively, were used for challenges. An additional isolate of ZIKV prototype strain MR766 with 150 suckling mouse brain passages and a single round of amplification on Vero cells was obtained from Robert Tesh (WRCEVA, Galveston, TX). After receipt, this virus also was amplified on C6/36 cells to produce stock virus (referred to as WRCEVA). See Table 1 for a full description of MR766 viruses.

**Table 1:** Summary of virus stocks and culture history. All Zika virus strains are the MR 766 prototype strain derived from the virus that was isolated from a sentinel rhesus monkey in Zika Forest, Entebbe, Uganda in April 1947[6]. All have undergone extensive mouse brain passage. The MR766 challenge stock was created for nonhuman primate natural history studies and was derived from the CDC virus. Challenge virus was prepared by inoculation of CDC virus onto a confluent monolayer of C6/36 mosquito cells and a clarified harvest of the culture medium was collected nine days post infection.

Stock Name	# of SM Passage	# Vero cell Passage	#C6/36 cell Passage
ZIKV MR766 WRCEVA	150	1	1
ZIKV MR766 CDC	149	2	0
ZIKV MR766 (challenge stock)	149	2	1

WRCEVA = World Reference Center for Emerging Viruses and Arboviruses at the University of Texas Medical Branch, CDC = Centers for Disease control and Prevention, SM = suckling mouse, Vero = African green monkey kidney cells, C6/36 = *Aedes albopictus* cells.

### Primary challenge

ZIKV MR766 challenge stock was thawed, diluted with PBS to the appropriate concentration for each challenge, and loaded into a 1mL syringe maintained on ice until challenge. For primary challenges, each of three, Indian-origin rhesus macaques was anesthetized and inoculated with 1mL subcutaneously over the cranial dorsum with either 1x10^4^, 1x10^5^, or 1x10^6^ PFU/mL of challenge stock. All animals were closely monitored by veterinary and animal care staff for adverse reactions and signs of disease. Animals were examined, and blood, pan urine, and oral swabs were collected from each animal daily from one through ten days post inoculation (dpi) and then weekly thereafter through 28 dpi. After 28 dpi, animals were rested for six weeks prior to secondary/heterologous challenge. Baseline sampling prior to secondary challenge occurred 56, 63, and 67 days post primary challenge.

### Secondary/heterologous challenge

Seventy days after primary challenge, ZIKV-FP was thawed and diluted with PBS to 1 x 10^4^ PFU/mL, loaded into a 1mL syringe and maintained on ice until challenge. Each animal was anesthetized and inoculated with 1mL subcutaneously over the cranial dorsum with 1x10^4^ PFU/mL ZIKV-FP. Animals were closely monitored by veterinary and animal care staff for adverse reactions and signs of disease. As described previously, animals were examined, and blood, urine, and saliva were collected from each animal daily from one through ten dpi and then weekly thereafter through 28 dpi.

### Plaque reduction neutralization test (PRNT90)

Macaque serum samples were screened for ZIKV neutralizing antibody utilizing a plaque reduction neutralization test (PRNT) on Vero cells (ATCC #CCL-81). Endpoint titrations of reactive sera, utilizing a 90% cutoff (PRNT90) were performed as described [23] against ZIKV-FP and challenge stock MR766.

### Viral RNA isolation

Plasma was isolated from EDTA-anticoagulated whole blood collected the same day by Ficoll density centrifugation at 1860 rcf for 30 minutes. Plasma was removed to a clean 15mL conical tube and centrifuged at 670 rcf for an additional eight minutes to remove residual cells. Urine was opportunistically collected from a pan beneath each animal’s cage and centrifuged at 500 rcf for five minutes to remove cells and debris. Saliva was collected using sterile oral swabs run under the tongue while animals were anesthetized. Swabs were placed in viral transport media (tissue culture medium 199 supplemented with 0.5% FBS and 1% antibiotic/antimycotic) for 60–90 minutes, then vortexed vigorously and centrifuged at 500 rcf for five minutes. Prior to extraction, swab samples were pelleted by centrifugation at 14000 rpm and 4°C for an hour. After centrifugation, supernatant was removed, leaving virus in 200 μL media. Viral RNA was extracted from 300 μL plasma or urine using the Viral Total Nucleic Acid Kit (Promega, Madison, WI) on a Maxwell 16 MDx instrument (Promega, Madison, WI). Viral RNA was extracted from 200 μL oral swab-derived samples using the QIAamp MinElute Virus Spin Kit (Qiagen, Germantown, MD) with all optional washes. RNA was then quantified using quantitative RT-PCR. Viral load data from plasma and urine are expressed as vRNA copies/mL. Viral load data from oral swabs are expressed as vRNA copies/mL eluate.

### Quantitative reverse transcription PCR (qRT-PCR)

For both ZIKV MR766 and ZIKV-FP, vRNA from plasma, urine, and oral swabs was quantified by qRT-PCR using primers with a slight modification to those described by Lanciotti et al. to accommodate the anticipated African ZIKV sequences [24]. The modified primer sequences are: forward 5’-CGYTGCCCAACACAAGG-3’, reverse 5’-CACYAAYGTTCTTTTGCABACAT-3’, and probe 5’-6fam-AGCCTACCTTGAYAAGCARTCAGACACYCAA-BHQ1-3’. The RT-PCR was performed using the SuperScript III Platinum One-Step Quantitative RT-PCR system (Invitrogen, Carlsbad, CA) on a LightCycler 480 instrument (Roche Diagnostics, Indianapolis, IN). The primers and probe were used at final concentrations of 600 nm and 100 nm respectively, along with 150 ng random primers (Promega, Madison, WI). Cycling conditions were as follows: 37°C for 15 min, 50°C for 30 min and 95°C for 2 min, followed by 50 cycles of 95°C for 15 sec and 60°C for 1 min. Viral RNA concentration was determined by interpolation onto an internal standard curve composed of seven 10-fold serial dilutions of a synthetic ZIKV RNA fragment based on ZIKV-FP. A comparison of the crossing point detected by qRT-PCR from the standard template, ZIKV-FP and ZIKV MR766 when using the universal primer set developed by our group suggests that efficiency of these primers is the same for both lineages of ZIKV and comparable to the efficiency of those designed by Lanciotti et al. for Asian ZIKV (S1 Fig).

### Deep sequencing

A vial of the same ZIKV MR766 stock used for primary challenge (i.e., challenge stock), a vial of the CDC MR766 stock, and a vial of the WRCEVA MR766 stock were each deep sequenced by preparing libraries of fragmented double-stranded cDNA using methods similar to those previously described [25]. Briefly, the sample was centrifuged at 5000 rcf for five minutes. The supernatant was then filtered through a 0.45-μm filter. Viral RNA was isolated using the QIAamp MinElute Virus Spin Kit (Qiagen, Germantown, MD), omitting carrier RNA. Eluted vRNA was then treated with DNAse I. Double-stranded DNA was prepared with the Superscript Double-Stranded cDNA Synthesis kit (Invitrogen, Carlsbad, CA) and priming with random hexamers. Agencourt Ampure XP beads (Beckman Coulter, Indianapolis, IN) were used to purify double-stranded DNA. The purified DNA was fragmented with the Nextera XT kit (Illumina, Madison, WI), tagged with Illumina-compatible primers, and then purified with Agencourt Ampure XP beads. Purified libraries were then sequenced with 2 x 300 bp kits on an Illumina MiSeq.

Virus populations replicating in plasma were sequenced using methods similar to those described previously [26]. Viral RNA was isolated from 500 μl of plasma using the QIAamp MinElute Viral RNA isolation kit, according to manufacturer’s protocol. Viral RNA was then subjected to RT-PCR using the Superscript III One-step RT-PCR kit (Invitrogen, Carlsbad, CA), MgSO4, and 1.2uM of the primer pairs ZUG-1F: TCAACAGATGGGGTTCCGTG; ZUG-1R: GGGGGAGTCAGGATGGTACT. The following cycling conditions were used: 55°C for 30 min; 94°C 2 min; 35 cycles of the following: 94°C 15 sec, 56°C 30 sec, and 68°C 3.5 min; 68°C 10 min. Viral cDNA amplicons were size selected by agarose gel electrophoresis and then purified using the Qiagen MinElute Gel Extraction kit. Purified PCR products were pooled and then ~1 ng of DNA was fragmented using the Nextera XT kit (Illumina), tagged with Illumina-compatible primers, and then purified with Agencourt Ampure XP beads. Purified libraries were then sequenced with 2 x 300 bp kits on an Illumina MiSeq.

Sequences were analyzed using a modified version of the viral-ngs workflow developed by the Broad Institute (http://viral-ngs.readthedocs.io/en/latest/description.html) and implemented in DNANexus. Briefly, host-derived reads that map to a human sequence database and putative PCR duplicates are removed. The remaining reads were mapped to an NCBI Genbank MR766 reference sequence (HQ234498). The published viral-ngs workflow uses the Novoalign read mapper; however, Novoalign is relatively insensitive to the 12 nucleotide in-frame deletion in the MR766 envelope. Therefore, we modified the viral-ngs pipeline to use the bwa mem version 1.5.0 (http://bio-bwa.sourceforge.net) read mapper with default parameters to map reads sequence reads to HQ234498. Deep sequencing datasets are available from http://go.wisc.edu/50bfn2 and are deposited in the NCBI Sequence Read Archive with accession numbers (SRP080884); the DNANexus workflow for read mapping is available upon request from the authors.

Mapped reads and reference scaffolds were loaded into Geneious Pro (Biomatters, Ltd., Auckland, New Zealand) for intrasample variant calling. Variants were called in the E protein that fit the following conditions: have a minimum p-value of 10e-60, a minimum strand bias of 10e-5 when exceeding 65% bias, and were nonsynonymous. Variant call format files are available from http://go.wisc.edu/50bfn2. Mapping metrics can be found in S2 Fig.

### Comparison of East African ZIKV MR766 and Asian Zika virus isolates

Full-length Asian-lineage Zika virus sequences available in NCBI Genbank as of June 8, 2016 were copied into Geneious Pro 9.1.2 (Biomatters, Ltd., Auckland, New Zealand). The amino acid sequence of the E protein was obtained from these sequences, as well as the consensus sequence from MR766 challenge stocks, by conceptual translation. These amino acid sequences were aligned with MUSCLE [27] as implemented in Geneious Pro 9.1.2 using default parameters.

#### Immunophenotyping

Numbers of activated and proliferating NK cells were quantified as described previously [28]. For each timepoint analyzed, 100 μL of EDTA-anticoagulated whole blood samples were incubated at room temperature for 15 minutes with an antibody master mix described in detail in Dudley et al. [22]. Red blood cells were lysed (BD Pharm Lyse, BD Biosciences, San Jose, CA), washed twice, and then fixed with 2% paraformaldehyde for 15 minutes. After fixation, cells were washed and permeabilized using Bulk Permeabilization Reagent (Life Technologies, Madison, WI) and stained with Ki-67 (clone B56, Alexa Fluor 647 conjugate) for 15 minutes. After staining, cells were washed again and resuspended in 2% paraformaldehyde until use in flow cytometry (BD LSRII Flow Cytometer, BD Biosciences, San Jose, CA). Flow cytometry data were analysed using FlowJo v. 9.9.3 (TreeStar, Ashland, OR).

#### PBMC processing

Fresh PBMC were isolated by Ficoll gradient as described in vRNA isolation. PBMC were collected into R10 media (Hyclone, Logan, UT) and centrifuged at 670 rcf for five minutes, treated with ACK (Grand Island, NY) to removed residual RBC, washed twice more with R10 media, and centrifuged again. R10 was removed and cells were resuspended in Cryostor CS5 media (BioLife Solutions, Bothell, WA), and frozen (1°C/ minute) down to -80°C storing in liquid nitrogen vapor phase until plasmablast assays were performed.

#### Plasmablast detection

PBMCs isolated from the three ZIKV-002 macaques were stained with the following panel of fluorescently labeled antibodies (Abs) specific for the following surface markers: CD20 FITC (L27), CD80 PE(L307.4), CD123 PE-Cy7(7G3), CD3 APC-Cy7 (SP34-2), IgG BV605(G18-145) (all from BD Biosciences, San Jose, CA), CD14 AF700 (M5E2), CD11c BV421 (3.9), CD16 BV570 (3G8), CD27 BV650(O323) (all from BioLegend, San Diego, CA), IgD AF647 (polyclonal)(Southern Biotech, Birmingham, AL), and HLA-DR PE-TxRed (TÜ36) (Invitrogen, Carlsbad, CA). LIVE/DEAD Fixable Aqua Dead Cell Stain Kit (Invitrogen, Carlsbad, CA) was used to discriminate live cells. Briefly, cells were resuspended in 1X PBS/1%BSA and stained with the full panel of surface Abs for 30 min in the dark at 4°C, washed once with 1X PBS, stained for 30 min with LIVE/DEAD Fixable Aqua Dead Cell Stain Kit in the dark at 4°C, washed once with 1X PBS, washed again with 1X PBS/1%BSA, and resuspended in 2% PFA Solution. Stained PBMCs were acquired on a LSRII Flow Analyzer (BD Biosciences, San Jose, CA) and the data was analyzed using FlowJo software v9.7.6 (TreeStar, Ashland, OR). Plasmablasts were defined similarly to the method previously described [29] excluding lineage cells (CD14+, CD16+, CD3+, CD20+, CD11c+, CD123+), and selecting CD80+ and HLA-DR+ cells (known to be expressed on rhesus plasmablasts and their human counterpart [30]).

## Results

### Sequence characterization of African lineage MR766 isolates

We elected to use the African ZIKV strain MR766 as the primary challenge virus since this is the prototypical East African ZIKV used in previous studies. MR766 was derived from the original isolate from the Zika Forest, Uganda [6]. Interestingly, GenBank contains records for seven different sequences all called ZIKV prototype strain “MR766” (accession numbers: DQ859059, AY632535, LC002520, KU963573, KU955594, KU720415, HQ234498). Differences among these MR766 sequences have been noted previously [31], but not extensively characterized. All of the Genbank sequences are 99.7–100.0% nucleotide identical to one another within the polyprotein coding sequence, with the exception of DQ859059. Others have shown that the sequence of DQ859059 matches a mosquito-derived sequence unrelated to MR766 [32]. This sequence should be considered a database error and not used as the prototype for any future MR766 analyses.

**Fig 1:**
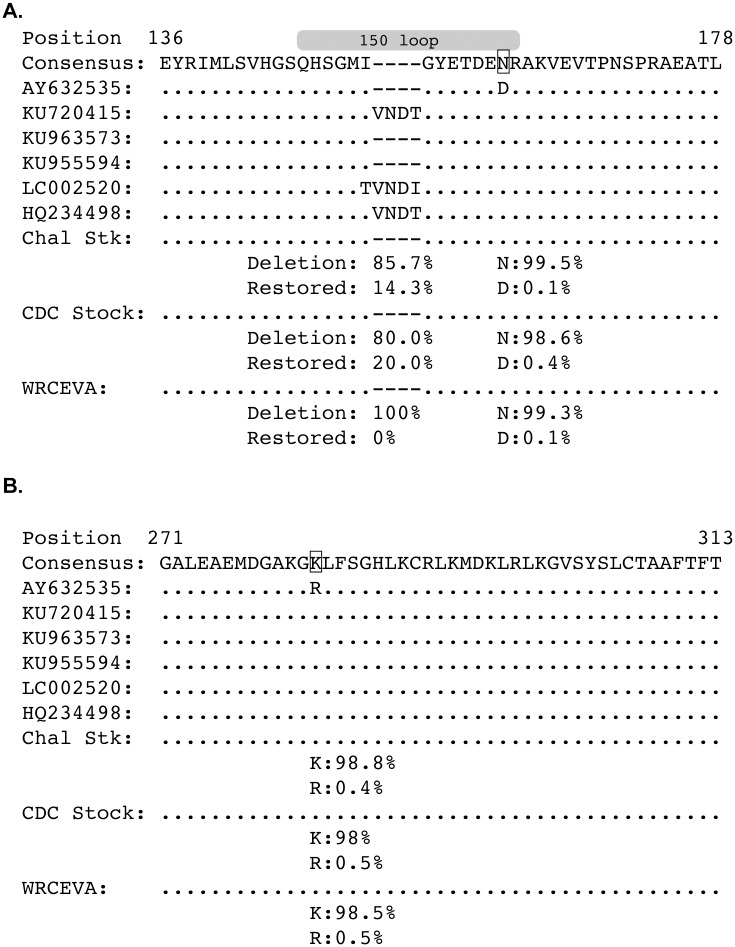
East African ZIKV MR766 envelope sequences often contain an in-frame deletion of an N-linked glycosylation site and are heterologous with respect to Asian ZIKV. The amino acid sequences of the Envelope protein for six ZIKV MR766 Genbank sequences were aligned to the consensus amino acid sequences of the three ZIKV MR766 stock viruses (Chal Stck, CDC Stock, and WRCEVA stock) using a Muscle alignment in Geneious. Dots represent identity to the consensus sequence. Dashes represent deletions. Only sections of the E protein with variations are shown, all other parts of the E protein showed no variation. Capital letters represent amino acids. The frequencies of the deletion and the restored deletion are shown below each of the stock sequences. Genbank reference sequence AY632535 had two amino acids that were different from the other reference sequences. The frequency of reads with these amino acid variants as determined by deep sequencing are shown below each of the stock sequences. **A.** Envelope protein amino acid region 136–178. The gray ellipse above the sequences represent the 150 loop of the E protein [20]. **B.** Envelope protein amino acid region 271–313.

The most obvious difference between the Genbank MR766 sequences is a four amino acid sequence in the ‘150 loop’ of the E protein that contains a potential N-linked glycosylation site [20] that is absent from some of the GenBank reference sequences; however, flavivirus E proteins are not universally glycosylated [33,34] and some strains of West Nile virus (WNV) contain the same four amino acid deletion that ablates E protein glycosylation [35]. Coincidentally, this WNV strain also originated in Uganda. We explored this deletion in greater detail by deep sequencing three MR766 isolates, Zika virus/R.macaque-tc/UGA/1947/MR766-3329 (hereafter referred to as challenge stock, or abbreviated Chal Stk in figures), WRCEVA, and CDC (see Table 1 for passage history). Fig 1A shows amino acid sites in the E protein that differ between the MR766 Genbank sequences and deep sequenced MR766 strains. The deletion was present in between 80.0% and 100.0% of sequencing reads from the three deep sequenced isolates. 85.7% of reads in the stock used to infect the animals in this study (i.e., ‘Chal Stk’ in Fig 1A) contained the deletion. There were two other amino acid sites that varied between the Genbank sequence AY632535 and the rest of the Genbank and stock consensus sequences. Both amino acid variants at each position were characterized in the deep sequenced strains and the frequency of each amino acid is shown in Fig 1A and 1B. In each stock, >98% of the sequence reads contained the amino acid found in the majority of Genbank sequences, while <1% of the sequence reads contained the amino acid found in AY632535.

Including the in-frame four-amino-acid deletion, MR766 is ~96% amino acid identical in the E protein to Asian ZIKV isolates (data not shown). In contrast, Asian ZIKV isolates are more than 99% amino acid identical to one another in envelope, typically differing by only 0–2 amino acids. In other words, the MR766 challenge stock is more genetically divergent from Asian ZIKV than Asian ZIKV strains are from one another. Therefore, we hypothesized that if immunity elicited by infection of macaques with MR766 protects against reinfection with Asian ZIKV infection, immunity elicited by any Asian ZIKV infection should be sufficient to confer complete protection against subsequent Asian ZIKV reinfection.

### Primary infection of Indian-origin rhesus macaques with East African Zika virus MR766

**Fig 2:**
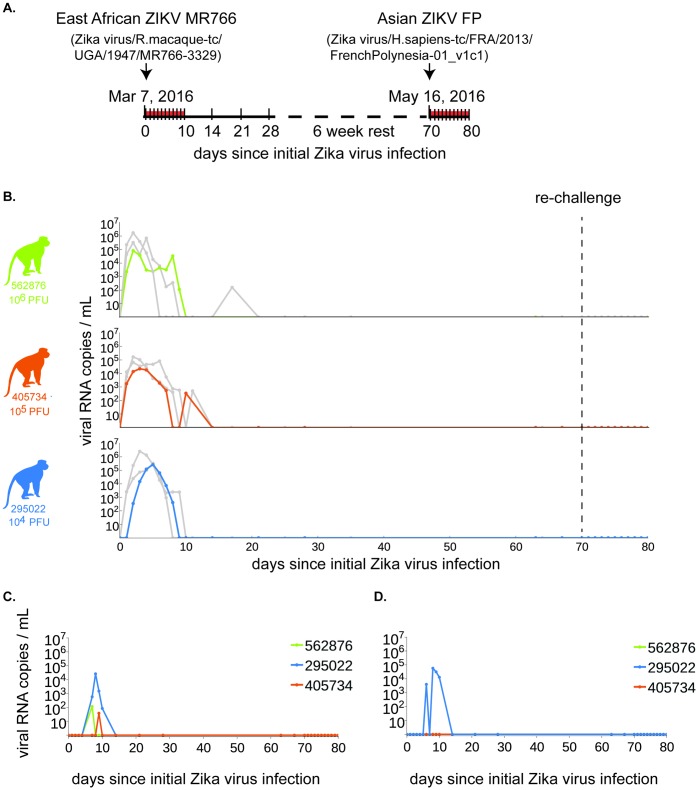
ZIKV-002 macaques challenged with ZIKV MR766 are protected from heterologous reinfection with ZIKV-FP. **A.** Study timeline with dates of primary and secondary, heterologous ZIKV challenges. Samples were collected daily from 0 to 10 dpi, and then weekly thereafter until secondary challenge (denoted by ticks along the timeline). Challenge stocks were derived from the East African and French Polynesian virus strains detailed above the timeline. **B.** Plasma viral loads, shown as vRNA copies/mL for each of the macaques challenged with 1 x 10^6^ (solid green line), 1x 10^5^ (solid orange line), or 1 x 10^4^ (solid blue line) PFU/mL of ZIKV MR766 challenge stock from the date of primary challenge through 10 days post heterologous challenge with ZIKV-FP. For comparison of plasma viral loads between ZIKV strains, solid light grey lines depict the plasma viral load trajectories for animals that were challenged with the same dose of ZIKV-FP and then rechallenged with homologous ZIKV-FP [22]. **C.** Oral swab and **D.** pan urine viral loads.

To examine the course of primary infection with MR766, we infected Indian-origin rhesus macaques (two males and one female). This group of animals was designated ZIKV-002 to provide consistency with real-time data on these animals that is publicly available at [36]. Animals were inoculated subcutaneously with either 1x10^4^, 1x10^5^, or 1x10^6^ PFU/mL of our challenge stock, consistent with the route and challenge doses of two previously published cohorts (ZIKV-001 and ZIKV-004) [22] of Indian-origin rhesus macaques challenged subcutaneously with Zika virus/H.sapiens-tc/FRA/2013/FrenchPolynesia-01_v1c1, an Asian ZIKV termed ‘ZIKV-FP’ for the remainder of the manuscript. A study schematic is shown in Fig 2A. All three animals were productively infected with MR766, with detectable plasma viremia one day post inoculation (dpi) in two of the three animals and in all three by two dpi using a qRT-PCR that amplifies MR766 and ZIKV-FP with essentially identical efficiency (S1 Fig). Plasma viremia peaked in all animals between two and five dpi, and ranged from 2.21 x 10^4^ to 2.64 x 10^5^ vRNA copies/mL. The highest plasma viremia was observed for the animal inoculated with the lowest primary challenge dose (1 x 10^4^ PFU/mL); the peak of plasma viremia also occurred later in this animal (five dpi) than in the other two. We postulate that this could be the result of high inoculating doses causing a rapid initial rise in viremia, which in turn induced a stronger innate immune response in these animals leading to quicker clearance of virus from the plasma, but confirmation will require further studies. By ten dpi, plasma viral loads were undetectable in all three animals. Plasma viremia is consistent with viremia of humans in the field as well as previous viremia found in macaques with an Asian-lineage virus [22,37]. Cerebrospinal fluid (CSF) was sampled at four dpi and 14 dpi, and was positive for vRNA in animal 295022 on day four (955 vRNA copies/mL) and 405734 on day 14 post-infection (937 vRNA copies/mL). 562876 was negative at both CSF collection timepoints. Detection of ZIKV RNA in other body fluids (saliva and urine) generally lagged behind detection in plasma by two to seven days. Viral RNA was detected in the saliva of two animals by seven dpi, in the third animal by nine dpi, and ranged from 3.8 x 10^1^ to 2.6 x 10^4^ vRNA copies/mL (Fig 2C). Viral RNA was detected in passively collected pan urine from only 295022 (Fig 2D). After 14 dpi, no animals had detectable vRNA in any body fluids at the remainder of the sampled timepoints.

### In-frame envelope deletion in MR766 stocks is rapidly lost in vivo

**Fig 3:**
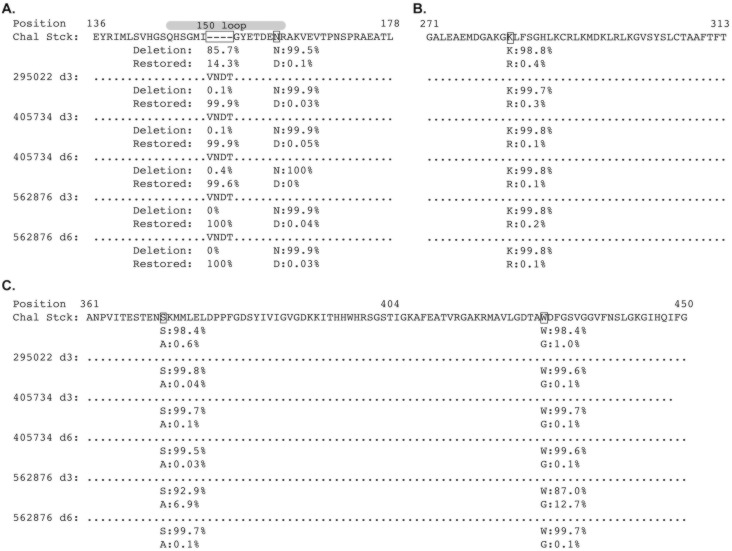
An N-linked glycosylation site in envelope is rapidly selected in vivo. Envelope sequences from the three animals were sequenced at three days post infection, and from two of the animals at day six post infection. A Muscle alignment of the translated sequences was generated in Geneious. Dots represent identity to the consensus sequence. Dashes represent deletions. Capital letters represent amino acids. Only regions of the E protein with sequence variants are depicted. **A.** E protein amino acid positions 136–178. The frequencies of the deletion and the restored deletion are shown below each of the stock sequences, with the indicated site boxed. Amino acid variant frequencies matching the variant sites in Fig 1A are shown. The gray ellipse above the sequence alignment represents the 150 loop of the E protein [20]. **B.** E protein amino acid positions 271–313. **C.** E protein amino acid positions 361–450. There were two additional nonsynonymous variants at greater than 5% in animal 562876 at day three, and the frequency of the amino acid variants from the other animals and time points are shown below each sample.

Three and six days after infection, vRNA from the animals was reverse transcribed and PCR-amplified using a primer pair that amplifies the E protein coding region, including the 12nt in-frame deletion in the 150 loop. PCR amplicons were randomly fragmented and deep sequenced. The in-frame deletion was detected in no more than 0.4% of all reads by three days post infection (Fig 3A). This suggests that the minority population in the challenge stock containing an intact 150 loop rapidly outcompeted viruses containing the in-frame deletion. Because the sequence of the intact sequence was identical to the sequence of the minority population of the challenge stock, *de novo* repair of the deletion by three days post-infection is unlikely. Some low level variation in other sites of the E protein were also seen relative to the challenge stock (Fig 3A, 3B and 3C), but all variants remained predominately the same as the challenge stock by six days post-infection.

### Robust cellular and humoral immunity to ZIKV

**Fig 4:**
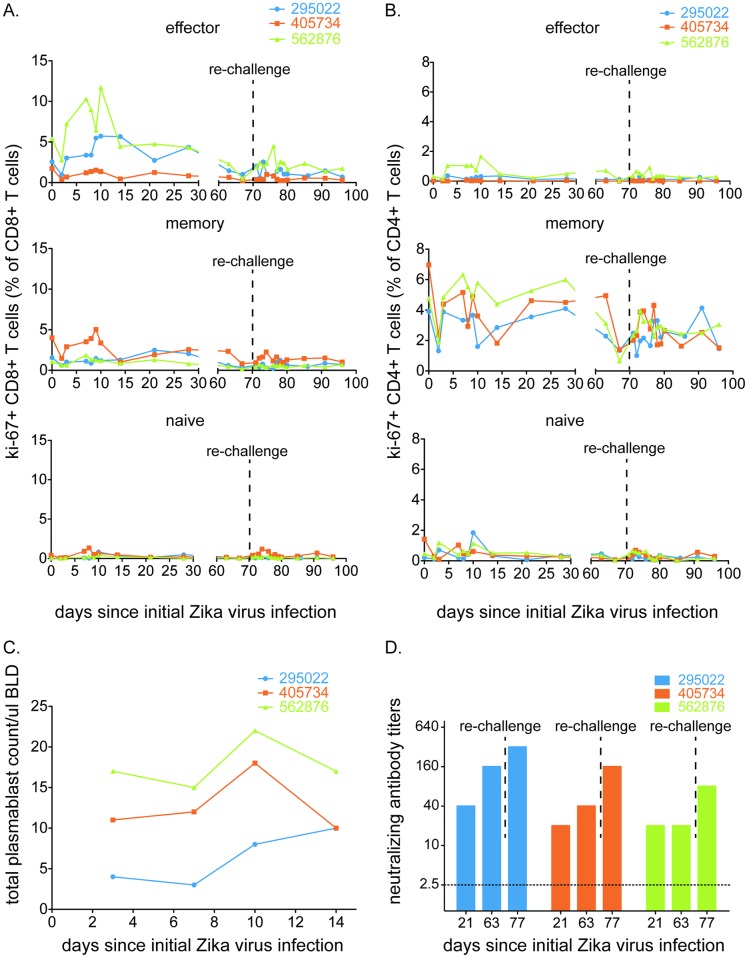
East African ZIKV MR766 infection elicits a robust, multifaceted immune response. Expansion of Ki-67+ (activated) **A**. CD8+ T cells (effector memory, central memory or naive) and **B.** CD4+ T cells (effector memory, central memory or naive) was measured at days 0, 2, 3, and days 7 through 10, then on days 14, 21 and 28 post-infection. After a rest period, activated immune responses were measured at days 63 and 67 post-infection. At 70 dpi, animals were heterologously re-challenged with ZIKV-FP. Expansion of activated cells was measured daily through 80 dpi, then at days 85 and 91 post-infection. Each population is presented as a percentage of the total CD8+ or CD4+ T cell population. **C.** Total number of plasmablast cells found in PBMCs collected at 3, 7, 10 and 14 dpi for each animal. **D.** PRNT_90_ titers 21 days post infection, 63 days post infection, and seven days post rechallenge for ZIKV-002 animals against ZIKV-FP. All three animals did not have detectable nAb titers prior to initial infection.

Proliferation of CD8+ and CD4+ T cells, as well as natural killer cells, was observed following ZIKV MR766 challenge (Fig 4A, 4B and S3 Fig). These responses, peaking six to ten days post challenge, were higher in the effector (CD95+, CD28-) and naive CD4+ and CD8+ T cell populations for most animals, while the memory (CD95+, CD28+) CD4+ and CD8+ T cells populations hovered around baseline values after an initial decrease at 2 dpi. The only exception was 562876, which proliferated above baseline values by 7 dpi. Re-challenge did not have a profound effect on any subset of T cell populations, but small increases in proliferating cells were observed in many CD4+ and CD8+ T cell populations in at least some animals. Ki-67+ NK cells peaked between 7 and 10 dpi and showed a modest increase after re-challenge (S3 Fig). Each animal also produced an interferon-gamma response to at least one ZIKV NS5 peptide as measured by whole PBMC ELISPOT (S4 Fig). The peak magnitude of proliferation was slightly lower, on average, than observed following ZIKV-FP infection but the small number of macaques in the study makes it impossible to quantify the significance of this observation. Similarly, we detected an increase in the number of antibody-producing plasmablasts in all three animals after MR766 infection (Fig 4C). Serum neutralizing antibody responses also were measured by plaque reduction neutralization tests (PRNT_90_), and all animals exhibited neutralizing antibody (nAb) titers ≥20 as early as 21 dpi (Fig 4D). Consistent with acute phase viremia data, the highest nAb titer was observed for the animal inoculated with the lowest primary challenge dose (1 x 10^4^ PFU/mL).

### Heterologous challenge with Asian ZIKV

To determine if primary infection with East African ZIKV results in protection from heterologous re-challenge with Asian ZIKV, we inoculated the ZIKV-002 animals with 1 x 10^4^ PFU/mL of ZIKV-FP at 70 dpi (10 weeks after primary challenge). This dose was chosen based on successful infection of 8/8 animals from multiple cohorts challenged with this dose to date ([22], zika.labkey.com). Viral RNA was undetectable in plasma (Fig 2B), saliva, and urine at all timepoints through 21 days post re-challenge. There was a significant increase in protection against infection with Asian ZIKV (Exact unconditional test) in animals previously infected with African ZIKV (n = 3) as compared to animals only challenged with Asian ZIKV (n = 8, *p* = 0.0016). All 11 animals were challenged with the same dose of the Asian ZIKV (1x10^4^ PFU/ml). However, we did observe an increase in nAb titers after re-challenge despite the lack of detectable viremia in the animals (Fig 4D) suggesting that low level virus replication did occur, indicating protection from disease rather than sterilizing immunity. Finally, nAb titer was similar when serum from the original challenge was screened against African versus Asian ZIKV (S5 Fig).

## Discussion

Here we describe the first evidence demonstrating that protective immunity resulting from natural ZIKV infection confers protection against detectable viremia following rechallenge with a heterologous genotype of the virus (African lineage followed by Asian lineage). This evidence is further supported by a recent study demonstrating that ZIKV likely circulates as a single serotype [38] and by a study that demonstrated protection with a heterologous strain of the virus, although both viruses were from the Asian lineage [39]. Together, these findings have potential implications for vaccine development and implementation. Although ZIKV immunology is still in its infancy, we hypothesize that, similar to other flaviviruses (e.g., Yellow fever virus (YFV), Japanese encephalitis virus (JEV), and Tick-borne encephalitis virus (TBEV)), nAbs are a critical component of the protective immune response [40–43]. In fact, for both JE and TBE vaccines a 1:10 PRNT50 nAb titer is regarded as protective in animals and humans [44,45]. Animals described here had nAb titers that far surpassed the 1:10 threshold (Fig 4D).

Like ZIKV, TBEV can be divided into three closely related subtypes- European, Siberian, and Far Eastern [46,47] but may also exist as even more distinct genotypes [48,49]. All three TBEV subtypes can and do co-circulate (e.g., in the Baltics) and data suggest that vaccines based on one subtype may also be effective against other subtypes and vice versa [42,50–53]. Similarly, YFV can be divided into seven major genotypes- five African and two South American [54] and it is generally accepted that strains of YFV constitute a single antigenic type (or serotype). However, differences in antigenicity between YFV strains, or between wild type and vaccine viruses, have been detected using mouse [55–60] and human [61] monoclonal and polyclonal antibodies. The continuing efficacy of YFV vaccines in reducing yellow fever disease in both Africa and South America suggests that the antigenic differences between individual YFV strains do not facilitate resistance to vaccine-induced immunity, i.e., diversity of YFV sequences appears to have little consequence for the use of currently formulated vaccines and/or the development of new YFV vaccines. Therefore, data from TBEV and YFV suggests that a vaccine made with any ZIKV genotype may be effective to protect against all genotypes, as demonstrated in our study between the Asian and Eastern African genotypes. Similar to TBEV, ZIKV and YFV, JEV exists as a single serotype [62,63] that is divided into five genotypes (GI-GV) [64,65]. All currently licensed JE vaccines are derived from GIII strains and are thought to be efficacious against all genotypes. It should be noted however that currently circulating JEVs display some degree of antigenic heterogeneity [66,67] and this antigenic heterogeneity has raised concerns about vaccine efficacy. For example, in mice, variation has been observed in the immunogenicity and protective efficacy of GIII JE vaccines against heterologous genotypes [63,68,69]. Lastly, like the other flaviviruses mentioned, nAbs also are thought to be an important component of the immune response to DENV [70,71], although the exact correlate of protection has yet to be elucidated. However, unlike the other flaviviruses, DENV exists as four distinct serotypes. Still, cross-serotype protection against symptomatic infection has been observed for up to two years after primary infection, after which point individuals were at greater risk of severe disease [72–74] because cross-serotype-reactive antibodies are believed to decay to sub-neutralizing levels that bind DENV without neutralization. Therefore, further work is needed (both experimental and epidemiologic) to understand the duration of protective ZIKV immunity after natural infection and to understand if any antigenic heterogeneity exists between the different lineages of ZIKV. Moreover, our study is limited in terms of sample size and because re-challenges were performed at only a single timepoint; we do not know how soon after primary infection protective immune responses emerge, or for how long they might endure. Nonetheless, there was significant protection (p = 0.0016) from infection displayed in animals previously infected with African ZIKV relative to animals not previously exposed to ZIKV.

Incidental to the primary observation of protective heterologous ZIKV immunity, we also demonstrated that selection appears to favor maintenance of four amino acids that are deleted in the majority of viral sequences in the challenge stock. It is interesting to note that this region contains a putative N-linked glycosylation site (E_154_), leading us to speculate that this might be consequential for *in vivo* replication. Flaviviruses contain several putative N-linked glycosylation sites (N-X-S/T) in the prM, E, and NS1 proteins. The glycosylation pattern on the viral envelope protein varies among flaviviruses and even among strains of the same virus [75–77], i.e., it is not universally glycosylated [33,34]. Some, but not all, African ZIKV strains contain a putative E glycosylation site at E_154_ [33]. This also is true of WNV E_154-156_ [78,79], DENV E_153_ and E_154_ [33], YFV E_155_ and E_158_ [59] and St. Louis encephalitis virus [80]. Still, N-linked glycosylation appears to play an important role in both the assembly and the infectivity of many flaviviruses [79,81–84]. For example, E protein glycosylation can influence virus infectivity for WNV [79,82,85]. Deglycosylation of both E and NS1 proteins of WNV completely attenuated neuroinvasiveness and induced protective immunity in the murine model with low doses of virus [86]. Likewise, deletion of the glycosylation site in the TBEV and WNV E protein resulted in substantially decreased viral particle release from mammalian cells [82,83], and DENV mutants lacking the glycosylation site at E_153_ were found to induce fusion at a higher pH than wild type DENV [84,87]. It has been hypothesized that extensive mouse brain or cell culture passage could lead to the deletion of the potential glycosylation site in ZIKV [88]. For example, it previously has been shown that extensive mouse brain and/or cell culture passage of DENV and YFV led to a progressive loss in pathogenicity for humans and increased neurovirulence in mice for these viruses [89–91], and most amino acid substitutions occurred in the structural protein genes [87,91]. Therefore, it is important to note that the African ZIKV strains analyzed here all underwent extensive mouse brain passage. Consequently, it will be important to sequence low passage, geographically distinct strains of the African lineage to confirm whether or not this glycosylation site polymorphism is an artifact of passage history or if it is representative of circulating strains in Africa. However, the fact that the deletion was likely selected against *in vivo* supports the hypothesis that passage history has influenced glycosylation sites in the African prototype strain and suggests that preservation of this glycosylation site may be important for efficient replication in primates. Given these results, caution should be taken when using the prototypical MR766 strain for vaccine, therapeutic, immunological or pathogenesis studies because its passage history may have altered the virus from what was originally circulating in Eastern Africa and what might productively infect and replicate in a human.

These results showing protection from heterologous virus have important implications for vaccine design and testing. MR766 is more genetically dissimilar to Asian lineage ZIKV isolates than any two Asian lineage ZIKV isolates are from one another. Unlike vaccines for other RNA viruses where immunogen selection is critical, our results suggest that protective immunity elicited against any Asian ZIKV should be sufficient to confer broad protection against all Asian ZIKV strains similar to what has been described for YFV, TBEV, and JEV. Together with our previous results, demonstrating that previous Asian-lineage ZIKV infection protects macaques from homologous rechallenge, the results shown here suggest that immunity elicited by a single ZIKV antigen may provide cross-protective immunity against a multitude of ZIKV strains.

The protection against detectable viremia from homologous and heterologous rechallenge also suggests that the immunologic barrier for complete protection may be comparatively low, such that vaccines with acceptable safety may have desirable efficacy even if they are not highly immunogenic. As recently published by Abbink, P. et al., multiple ZIKV vaccine modalities have been successful in rhesus macaques and will likely be effective in humans [92]. We do not currently know the exact correlate of protective immunity in these animals, since robust memory T cell, NK cell, B cell, and nAb responses were elicited, but nAbs alone appeared to be effective in ZIKV vaccine studies in macaques [92].

## Supporting Information

S1 Fig Validation of Universal Primers by qRT-PCR. Crossing point indicates threshold PCR cycle at which amplification was first detected. **A.** Comparison of crossing points seen in amplification of a synthetic ZIKV-FP standard curve using our universal primers and those designed by Lanciotti et al [24]. **B.** Comparison of amplification efficiencies of universal primers for East African MR766 and ZIKV-FP targets. Universal primers were used in qRT-PCR to amplify serial tenfold dilutions of MR766 or ZIKV-FP stocks. Amplification efficiencies were 1.975 and 1.956 for MR766 and ZIKV-FP, respectively, with 2 being a theoretically perfect efficiency (i.e., DNA concentrations double each cycle).
(TIF)

S2 Fig Metrics for deep sequencing of virus stocks and samples from animals. **A**. Metrics for the sequences of the three stocks (Fig 1A) spanning the *env* gene are shown. The number of individual reads mapping to *env* and the average depth of coverage are shown. **B**. Metrics for the *env* sequences generated from animals (Fig 3) are shown. Theoretical number of templates was calculated by assuming that isolation of viral RNA from 500μl of plasma was complete into 25μl of elution buffer, and was followed by using 3μl of eluted viral RNA per RT-PCR reaction. The number of individual reads mapping to *env* and the average depth of coverage are shown.
(TIF)

S3 Fig Ki-67+ NK cell proliferation. Ki-67+ NK cells presented as the % of total NK cells for each animal through both the first ZIKV challenge (ZIKV MR766) and re-challenge (ZIKV-FP) with a heterologous virus.
(TIF)

S4 Fig Antigen-specific T cell responses by IFNg-ELISPOT. PBMC was stimulated with peptide pools spanning the African lineage NS5 peptide (GenBank: DQ859059) at the 4dpi, 10dpi, and 14dpi time points. Concanavalin A (ConA) was used as a positive control. Each sample was run in duplicate. **A.** Data were baseline corrected by subtracting the average negative control values from each response. A threshold of 10.0 SFC/100,000 cells was set as the minimum value to be considered a positive T cell response, as indicated by the dashed line. **B.** Each pool was comprised of 10 overlapping 15mer peptides offset by 4 amino acids. **C.** Several peptide pools elicited T cell responses at multiple time points in multiple animals with shared MHC haplotypes, suggesting T cell responses are restricted by MHC alleles.
(TIF)

S5 Fig Neutralizing antibody titers. PRNT90 titers seven days prior to rechallenge for ZIKV002 animals against Asian ZIKV FP (open bars) and East African ZIKV MR766 (filled bars).
(TIF)
