# Supplementary figures and images for: Heterologous Protection against Asian Zika Virus Challenge in Rhesus Macaques
Source: PLoS Negl Trop Dis. 2016 Dec 2;10(12):e0005168. doi: 10.1371/journal.pntd.0005168 (PMC5135040; doi:10.1371/journal.pntd.0005168)

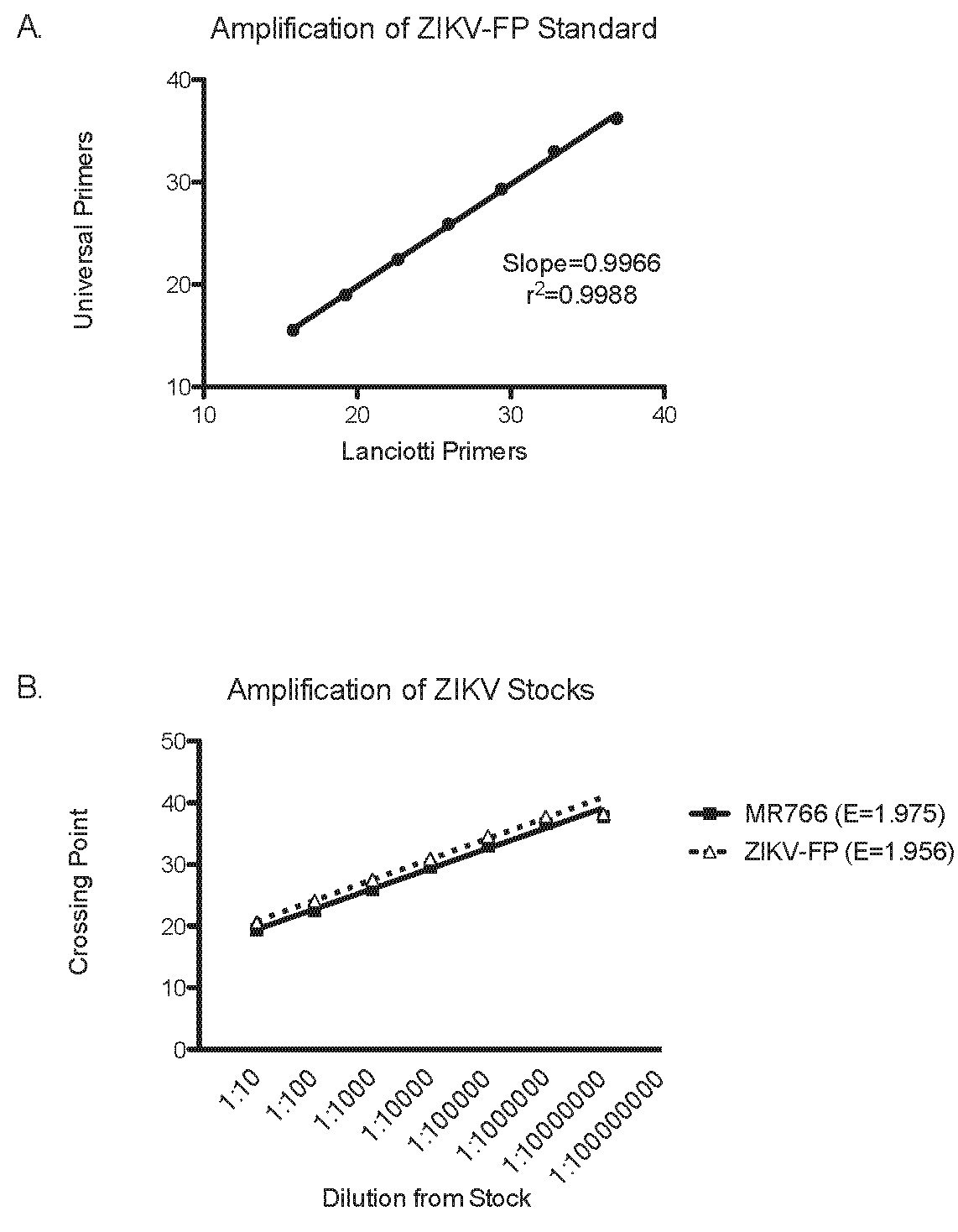

Supplement: S1 Fig — Crossing point indicates threshold PCR cycle at which amplification was first detected. A. Comparison of crossing points seen in amplification of a synthetic ZIKV-FP standard curve using our universal primers and those designed by Lanciotti et al [24]. B. Comparison of amplification efficiencies of universal primers for East African MR766 and ZIKV-FP targets. Universal primers were used in qRT-PCR to amplify serial tenfold dilutions of MR766 or ZIKV-FP stocks. Amplification efficiencies were 1.975 and 1.956 for MR766 and ZIKV-FP, respectively, with 2 being a theoretically perfect efficiency (i.e., DNA concentrations double each cycle). (TIF) [file pntd.0005168.s001.tif]

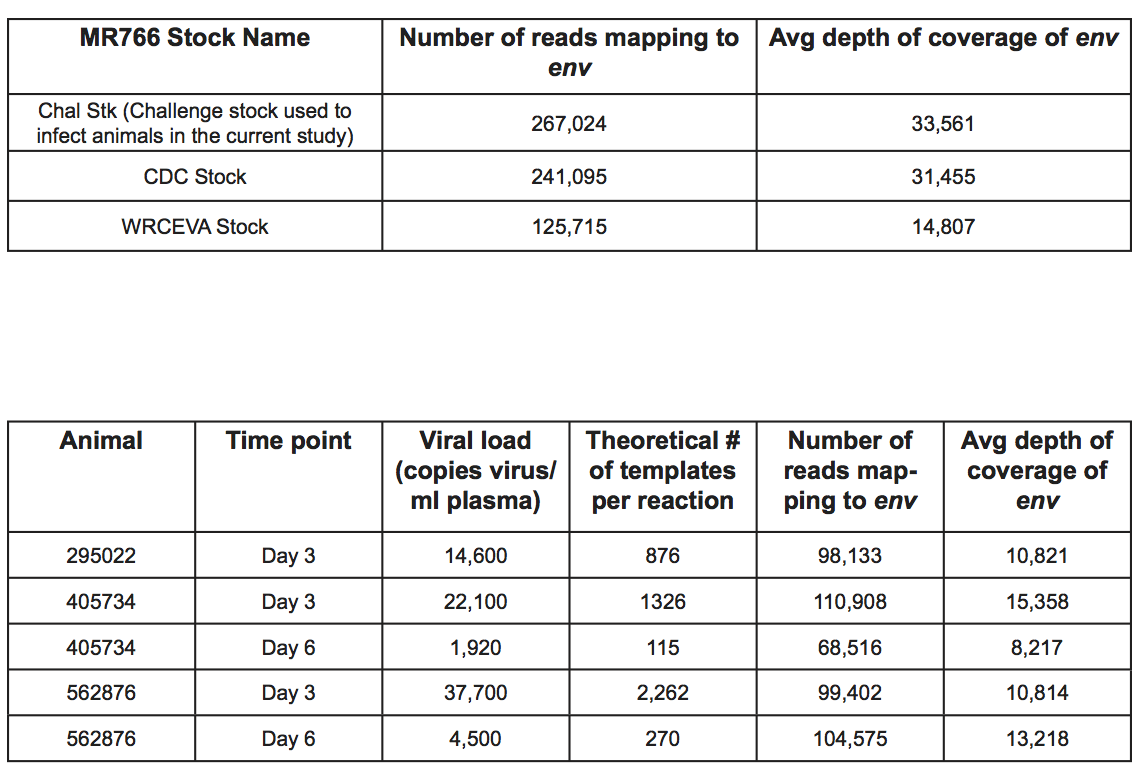

Supplement: S2 Fig — A. Metrics for the sequences of the three stocks (Fig 1A) spanning the env gene are shown. The number of individual reads mapping to env and the average depth of coverage are shown. B. Metrics for the env sequences generated from animals (Fig 3) are shown. Theoretical number of templates was calculated by assuming that isolation of viral RNA from 500μl of plasma was complete into 25μl of elution buffer, and was followed by using 3μl of eluted viral RNA per RT-PCR reaction. The number of individual reads mapping to env and the average depth of coverage are shown. (TIF) [file pntd.0005168.s002.tif]

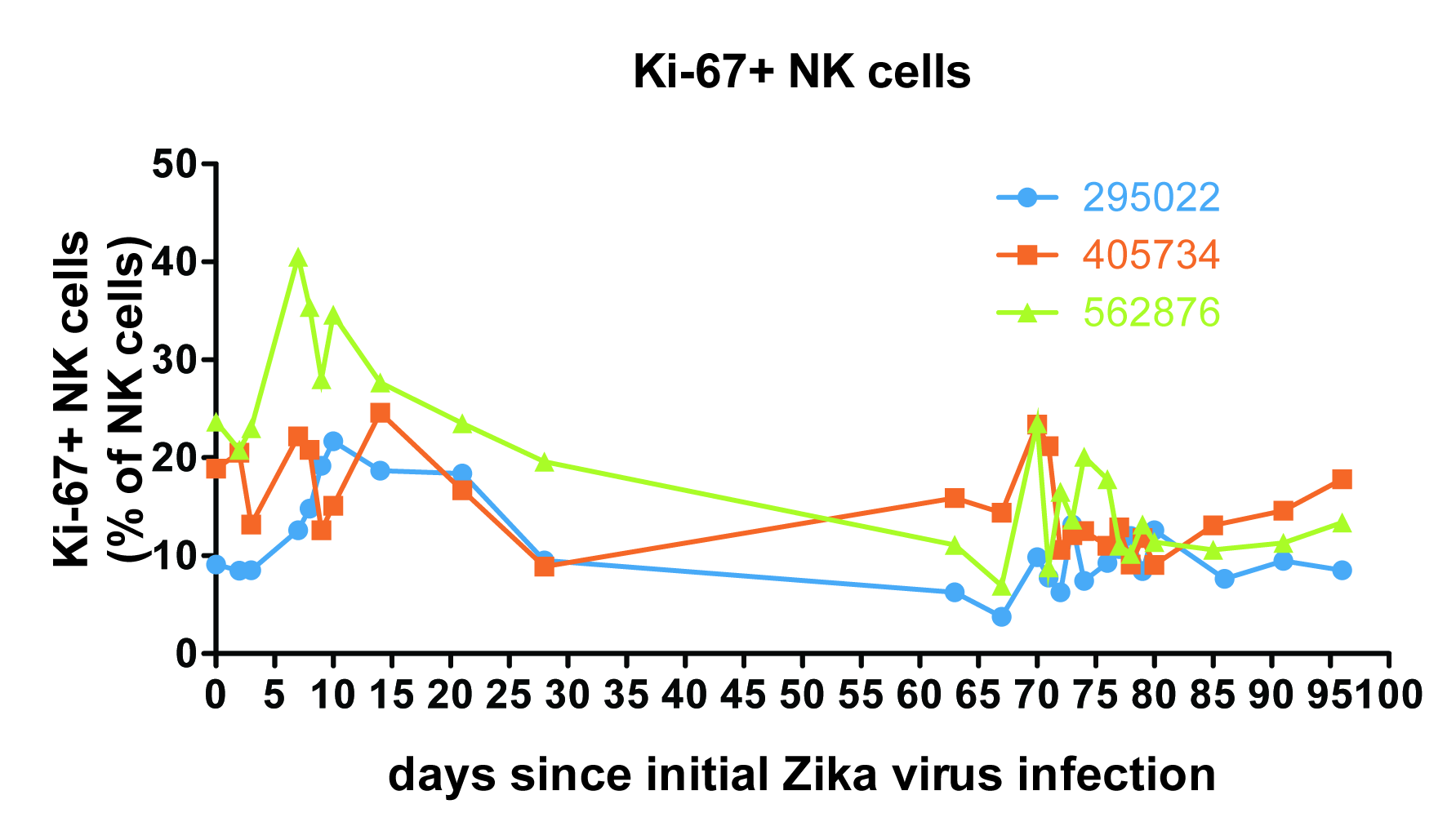

Supplement: S3 Fig — Ki-67+ NK cells presented as the % of total NK cells for each animal through both the first ZIKV challenge (ZIKV MR766) and re-challenge (ZIKV-FP) with a heterologous virus. (TIF) [file pntd.0005168.s003.tif]

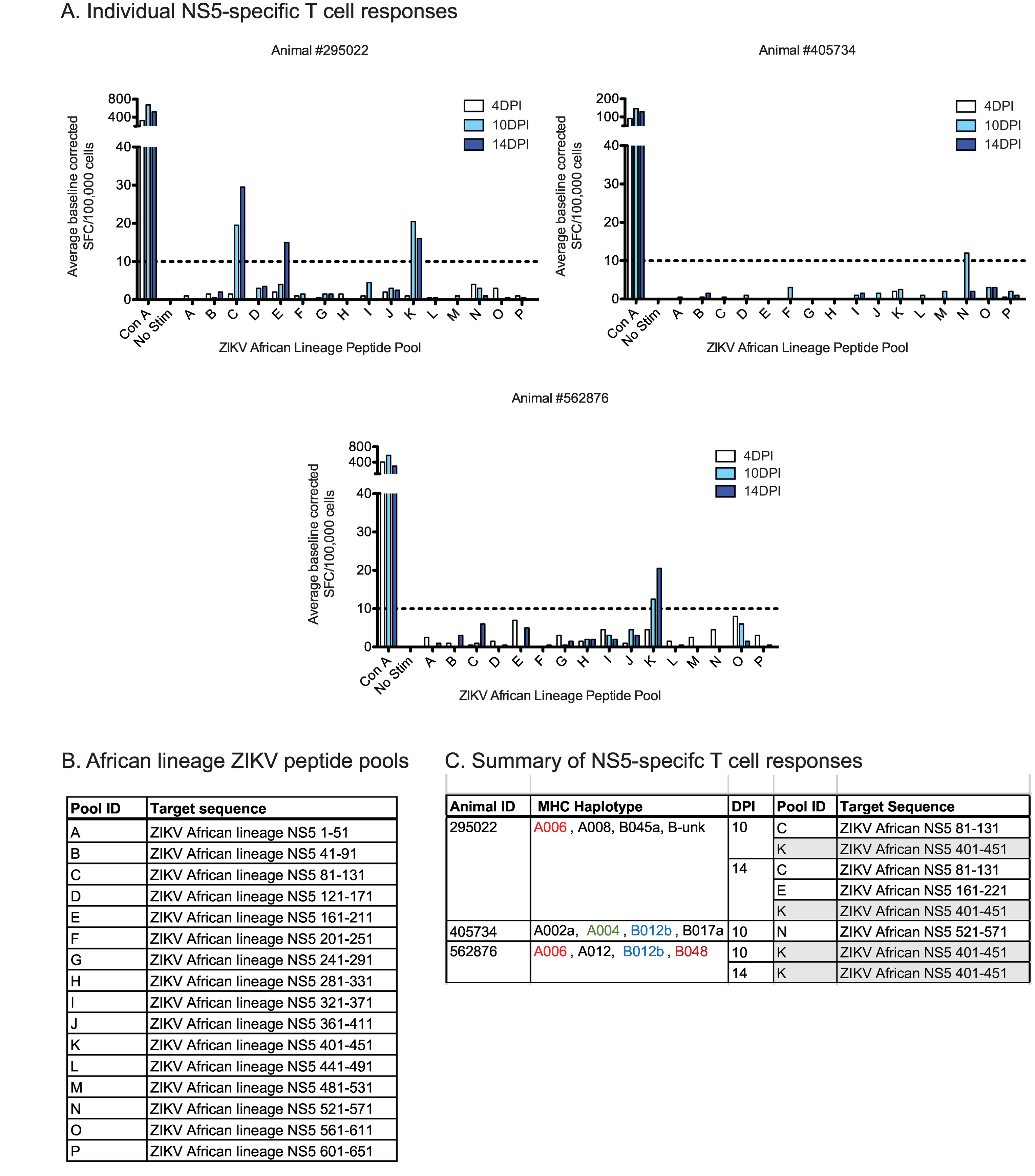

Supplement: S4 Fig — PBMC was stimulated with peptide pools spanning the African lineage NS5 peptide (GenBank: DQ859059) at the 4dpi, 10dpi, and 14dpi time points. Concanavalin A (ConA) was used as a positive control. Each sample was run in duplicate. A. Data were baseline corrected by subtracting the average negative control values from each response. A threshold of 10.0 SFC/100,000 cells was set as the minimum value to be considered a positive T cell response, as indicated by the dashed line. B. Each pool was comprised of 10 overlapping 15mer peptides offset by 4 amino acids. C. Several peptide pools elicited T cell responses at multiple time points in multiple animals with shared MHC haplotypes, suggesting T cell responses are restricted by MHC alleles. (TIF) [file pntd.0005168.s004.tif]

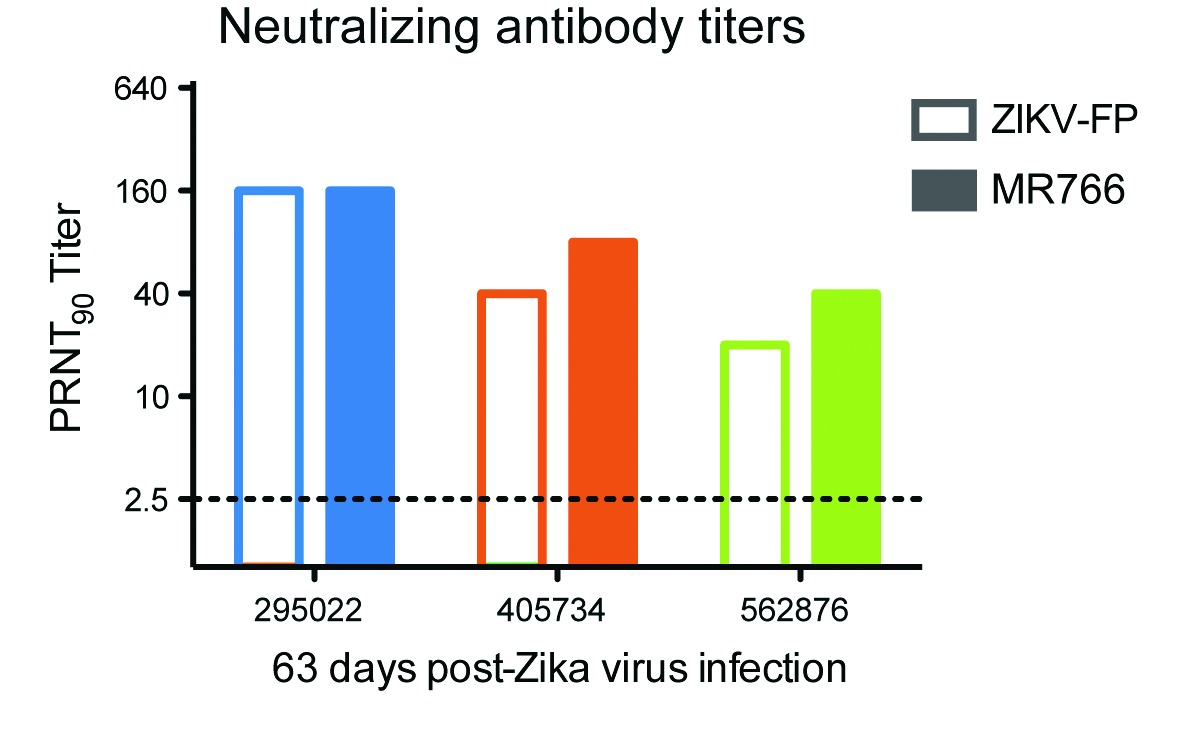

Supplement: S5 Fig — PRNT90 titers seven days prior to rechallenge for ZIKV002 animals against Asian ZIKV FP (open bars) and East African ZIKV MR766 (filled bars). (TIF) [file pntd.0005168.s005.tif]
